# Challenges to Physical Activity Participation Among Older People Living with HIV: Scoping Review

**DOI:** 10.3390/ijerph22101513

**Published:** 2025-10-02

**Authors:** Nongiwe Linette Mhlanga, Sikhumbuzo A. Mabunda

**Affiliations:** 1 The Department of Public Health, Walter Sisulu University; Mthatha 5117, South Africa; 2 School of Population Health, University of New South Wales, Sydney 2052, Australia; 3 The George Institute for Global Health, University of New South Wales, Sydney 2052, Australia

**Keywords:** physical activity, exercise, older people, human immunodeficiency virus, challenges

## Abstract

Older people living with Human Immunodeficiency Virus (OPLWH) have lower physical activity levels than other population groups. Inadequate physical activity participation increases the risks of age-related non-communicable diseases (NCDs). Therefore, this scoping review aimed to describe the challenges experienced by OPLWH in physical activity participation. The Joanna Briggs Institute (JBI) framework for conducting scoping reviews was used. Articles that described physical activity among OPLWH in all contexts, were written in English, and published between 2015 and 2025 were included. Searches were conducted from the 23 January 2025 from Google Scholar, PubMed, and Scopus using the following keywords: challenges, older, HIV, and physical activity. Two reviewers selected studies against the eligibility criteria, and disagreements were discussed. Data were extracted using Microsoft Excel and analysed using content analysis. A total of 1291 studies were screened by title and abstract, and 17 were included. Most (35.3%, n = 6) were from the United States of America. Two themes emerged: individual-related challenges and community or environmental-related challenges. Individual-related challenges include poor physical health, pain, depression, and lack of motivation. Community or environmental-related challenges were HIV-related stigma, environmental safety concerns, and negative gym experiences. The review was limited by a low inclusion of sub-Saharan African countries, affecting the generalisability of its findings to this region.

## 1. Introduction

Physical activity among older people living with Human Immunodeficiency Virus (HIV) is an essential self-care activity for mitigating risks of developing age-related chronic illnesses [1]. For it to be beneficial, it should be of adequate intensity and regularity [2]. However, ageing with HIV has been associated with low physical activity levels [3,4]. Notably, older people living with HIV (OPLWH) are often defined as those aged 50 years and above [5]. In the United States of America (USA), OPLWH spend 75% of their awake time sedentary, with only 24% performing low-intensity physical activity [6]. This could be attributed to the combined effects of loss of muscle mass and function due to natural physiological processes of ageing and chronic inflammation from HIV and antiretroviral therapy (ART) toxicity [7]. Although OPLWH have lower levels of physical activity, people generally have different levels of physical activity, which vary daily [8].

Physical activity refers to bodily movements the skeletal muscles make, resulting in energy expenditure [9]. It subsumes exercise, which is structured, iterative, and planned to ensure physical fitness [9]. Coupled with dietary interventions, physical activity is safe [10], reduces the risks of cardiometabolic conditions [11,12,13], and improves cardiorespiratory function [10] and mental health [14]. Although studies [10,11,12,13,14] outline these benefits, a systematic review found that no significant benefits existed between physical activity performance, on one hand, and weight reduction, cardiovascular risk, or health-related quality of life on the other [14]. Such contrasting views, however, do not negate the finding that OPLWH have lower levels of physical activity and face challenges in engaging in physical activity. These challenges are in initiating, performing, and sustaining adequate physical activity [15,16,17]. An example is pain, which older people who are HIV-negative also experience [18], although a larger proportion of OPLWH experience chronic pain [19]. This may be because of HIV and or ART toxicity on the central and peripheral nervous systems, combined with comorbidities of ageing that cause pain such as osteoarthritis [20].

In reference to ageing among PLWH, the word “older”, although referring to persons aged 50 and above [5], bares added significance in this context as HIV infection management has changed over the past four decades due to advancements in treatment, with the life expectancy of PLWH closely mirroring HIV-negative people [21]. Illustrating this overall well-being of PLWH, in Ivory Coast, differences in physical activity performance between PLWH and HIV-negative people only appear after the age of 60 [7]. Nonetheless, in this study, the age of 50 years and above was used to indicate “older” persons.

OPLWH are a heterogeneous population that varies in age, gender, residency (urban or rural), culture, socioeconomic status, and type and/or presence of comorbidities. These differences affect predisposition to challenges in physical activity participation. Considering context differences, for example, in the USA and Cameroon, older women living with HIV are less physically active than their male counterparts [22,23]. In contrast, in rural Uganda, there was no significant difference between older men and women [24]. In addition, there are differences in HIV and ART-related factors like viral load, duration of ART use, and HIV infection and the presence of Acquired Immunodeficiency Syndrome (AIDS)-defining illnesses. These HIV and ART factors correlate with physical activity levels. For example, the long duration of HIV infection is associated with poor physical function [25].

The geographical or regional context also warrants special attention, as HIV prevalence is highest in sub-Saharan Africa, which bears at least two-thirds of the global HIV burden [26]. By 2020, at least 80% of all OPLWHs lived in low to middle-income countries (LMICs) in Eastern and sub-Saharan Africa [27]. While prevalence is low, the proportions of OPLWH in high-income countries are higher than in LMICs [27]. These differences in the geographical distribution of OPLWH require the promotion of adequate physical activity levels through mitigating identified barriers and challenges, especially in sub-Saharan Africa. This could enhance the benefits of reduced cardiometabolic risks, improved cardiorespiratory function, and mental health [10,11,12,13,14] among OPLWH in these regions.

The World Health Organisation (WHO) Guidelines for Physical Activity and Sedentary Life (2020) outline what constitutes adequate levels of physical activity [2]. The guidelines stipulate that older people with chronic conditions should perform moderate-intensity aerobic physical activity for 150 to 300 min weekly or vigorous-intensity aerobic exercise for 75–150 min weekly [2]. Alternatively, a combination of moderate and vigorous-intensity physical activity can be performed [2]. Muscle strengthening exercises for all muscle groups should be performed for at least two days a week [2]. National physical activity plans have been developed to promote physical activity levels, with at least 80% of countries in the WHO European network having physical activity guidelines by 2016 [28]. In sub-Saharan Africa, the WHO Africa region adopted the Global Action Plan on Physical Activity (GAPPA) 2018–2030, which aims to reduce physical inactivity by 15% by 2030 [29]. Paradoxically, few studies describe the challenges OPLWH experience in physical activity participation in sub-Saharan Africa [25].

Some studies [3,30] synthesised evidence on correlates of physical activity participation among PLWH (younger and older). Other studies have used original research designs to describe the challenges faced by OPLWH in physical activity participation [15,31]. However, gaps remain in evidence synthesis focused solely on OPLWH. Thus, this scoping review fills two research gaps: (1) a gap in population, where there is an underrepresentation of OPLWH in evidence synthesis studies, and (2) a methodological gap where previous studies have applied empirical research designs. Outlining barriers to physical activity participation will assist in developing policies and programmes that include measures to address them. A preliminary PubMed and Google Scholar database search was conducted, and no similar scoping reviews were found. Therefore, the review aimed to answer the following research questions: What are the challenges experienced by OPLWH in participating in physical activity? It also looked to address the following secondary questions: What are the characteristics of OPLWH who experience challenges in physical activity participation? Where do OPLWH experience challenges in physical activity participation?

## 2. Materials and Methods

The protocol for this scoping review was registered on the Open Science Framework, registration number https://osf.io/td8qy/ accessed on 23 June 2025. The Joanna Briggs Institute (JBI) framework for conducting scoping reviews, aligned to the Preferred Reporting Items for Systematic Reviews (PRISMA), was used [32,33]. Searches for relevant sources were performed from the 23 January 2025. Database searching was conducted in consultation with a Walter Sisulu University librarian. Three databases—Scopus, Google Scholar, and PubMed—were searched. The database search used the broad key terms older, HIV, physical activity, and challenges. The full search strategy is included as Additional File S1.

Covidence was used to screen the studies using set eligibility criteria. The inclusion and exclusion criteria were delineated in alignment with the population, concept, and context (PCC) framework. Therefore, the review included studies whose population comprised OPLWH where the mean/median/average ages of PLWH were 50 or more years, where challenges were experienced in any setting. The types of articles included were those published from 2015 to 2025 to maintain relevance and those published in English. We excluded grey literature such as book chapters, institutional reports, dissertations, editorials, and conference proceedings, as some may not have been based on empirical research and because of difficulties in assessing their quality. This exclusion may affect the breadth of included literature and the findings. We also excluded articles not published in English to minimise translation bias.

After screening, the Mendeley reference manager was used to remove duplicates. Articles were screened by reading the titles and abstracts. Full texts that appeared to meet the inclusion criteria were then read for final selection. Two reviewers screened articles for selection, and any disagreements were discussed until consensus was reached.

The selected studies were assessed for risk of bias using the JBI critical appraisal tools by two reviewers. The appraisal was performed independently, and any differences were resolved through discussion. Three JBI appraisal tools used included the qualitative checklist [34], cohort checklist [35], and checklist for analytical cross-sectional studies [35]. The checklists assessed studies for the quality of the methodology and the extent to which bias was addressed in the research design [34,35]. To appraise the studies, the researchers responded to criteria with responses of “yes”, “no”, “unclear”, and “not applicable” across the three checklists used. The overall appraisal decision from these criteria was categorised into “include”, “exclude”, or “seek further information”.

After the appraisal, data from the selected studies were charted using Microsoft Excel (Additional File S2). The researchers developed this Microsoft Excel chart guided by the JBI framework [33]. The following data were extracted: database retrieved from, title, objectives, country of origin, authors, year published, purpose, research design, setting, and sample characteristics (sample size, ages, presence of comorbidities, and mean duration of HIV infection). Findings relevant to the review questions were also extracted.

A descriptive numerical analysis of the studies was performed using Microsoft Excel, where the years of publication and the geographical context were analysed. Qualitative content analysis was used to answer the research questions. This analysis method was selected as it can be applied to qualitative and quantitative data to derive patterns and associations in text [36]. Four steps of identifying units with similar meanings, labelling with codes, grouping the codes into categories, and describing the categories with a theme were used [36].

## 3. Results

### 3.1. Search Results

**Figure 1:**
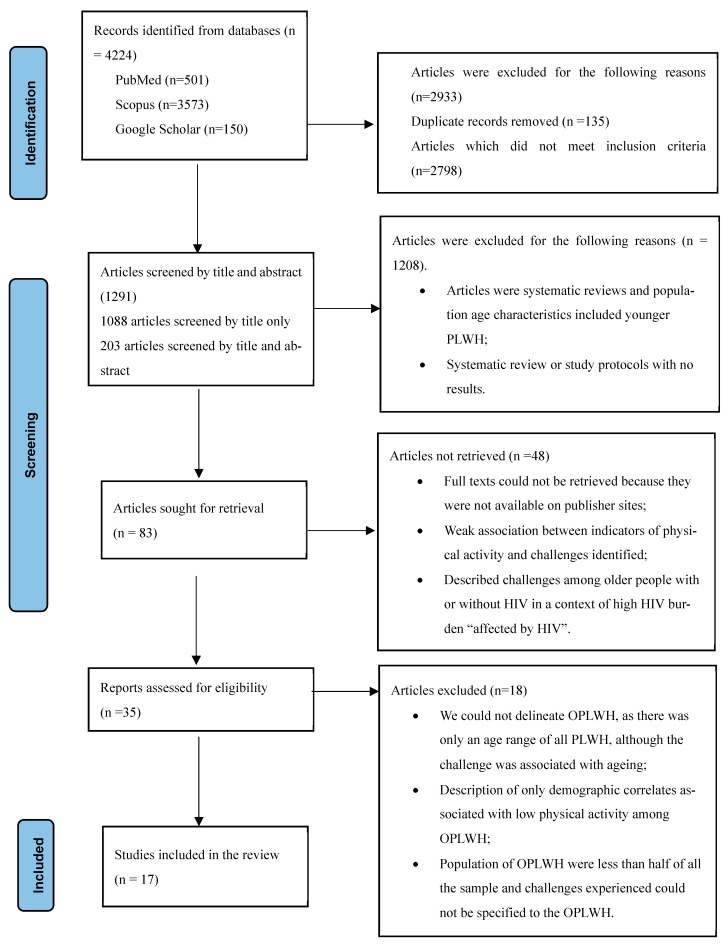
PRISMA flow chart to show the decision process for study selection. Source: adapted from Page et al. [37].

From the methods described above, we removed duplicate and other articles that did not meet the inclusion and exclusion criteria, and 1291 articles were screened by title and abstract. Of these, 83 were sought for retrieval and 35 were assessed for eligibility, with a final selection of 17 studies included in the review. Figure 1, which follows, illustrates the PRISMA flow chart of the decision process in study selection.

### 3.2. Characteristics of Selected Studies

The review comprised 17 studies drawn from 2016 to 2023. Most (35.3%, n = 6) studies were from the USA, and five (29.4%) were from Canada. One (5.9%, n = 1) study each was drawn from Uganda, China, the United Kingdom (UK), South Africa, and Cameroon, while one (5.9%) study was drawn from two countries, Côte d’Ivoire and Senegal. Regarding the year of publication, most (23.5%, n = 4) studies were from 2021, three (17.6%) studies each were published in 2022 and 2023, followed by 2019 and 2017, when two (11.8%) selected studies were published. One (5.9%) study each was published in 2016, 2018, and 2020. The selected articles used various designs; most studies (52.9%, n = 9) used a qualitative approach. Table 1 summarises the characteristics of the studies reviewed.

**Table 1:** Characteristics of the studies reviewed.

Author and Year	Study Origin	Study Approach	Sample Characteristics	Purpose of Study	The Outcome in Relation to the Scoping Review
Johs et al. (2019) [16]	USA	Qualitative study using focus group discussions.	29 PWLH; median age women 56, men 57 years.	To describe barriers and benefits to exercise among OPLWH.	Negative gym experiences. Lack of motivation. Poor health and multimorbidity.
Li et al. (2023) [38]	USA	Quantitative study.	174 PLWH Mean 51.3 years.	To assess factors associated with physical activity performance among PLWH and HIV-negative controls.	Depression.
Derry-Vick et al. (2022) [39]	USA	Quantitative study using biomedical measures of physical function, depression, pain, and inflammatory markers.	160 OPLWH Mean age 61 years.	To investigate cross-sectional assessments between pain, physical function, and inflammatory markers.	Pain.
Nguyen et al. (2017) [40]	USA	Qualitative study using four focus group discussions.	27 OPLWH Mean age 54 years.	To seek feedback from a community-based exercise intervention programme.	Pain. Poor health and multimorbidity. Negative gym experiences.
Simonik et al. (2016) [41]	Canada	Descriptive qualitative study.	14 PLWH Median age 50 years.	To assess readiness to engage in exercise among PLWH with multimorbidity.	Pain. Depression. Poor health and multimorbidity. Negative gym experiences.
Solomon et al. (2021) [42]	Canada	Longitudinal qualitative study.	11 OPLWH Median age 52 years.	To assess the experiences of PLWH who engaged in a physical activity programme.	Poor health and multimorbidity. Negative gym experiences.
Bernard et al. (2021) [25]	Cote d’Ivoire and Senegal	Cross-sectional analytical study.	333 OPLWH Median age 57 years.	To describe the prevalence of physical function and factors associated with poor physical function impairment among OPLWH.	Poor health and multimorbidity.
Montgomery et al. (2017) [43]	Canada	Descriptive qualitative study.	11 PLWH median age 52 years.	To explore the experiences of PLWH engaged in a community-based exercise programme.	Poor health and multimorbidity.
Homayouni et al. (2021) [44]	Canada	Descriptive qualitative study	10 OPLWH median 58 years	To explore experiences of a physiotherapy-led exercise programme among PLWH.	HIV-related stigma. Poor health and multimorbidity. Lack of motivation. Negative gym experiences.
Qin et al. (2022) [45]	China	Cross-sectional study	315 OPLWH Mean age 61.3 years	To compare neurocognitive performance between OPLWH and HIV-negative controls, and assess if HIV neurocognitive function was mediated by depression and physical activity.	Depression.
Tatsilong Pambou et al. (2023) [22]	Cameroon	Cross-sectional study	136 OPLWH mean age 57.07	To determine levels of physical activity and quality of life, and to determine the extent to which quality of life domains influence physical activity.	Depression. Poor physical health and multimorbidity.
Duncan et al. (2020) [11]	UK	Mixed methods study	23 PLWH Average 54 years	To describe the feasibility and acceptability of an individualised diet and physical activity intervention to reduce type 2 diabetes among PLWH.	HIV-related stigma. Lack of motivation. Negative gym experiences.
Wright et al. (2021) [31]	Uganda	Mixed, observational study	30 PLWH Mean age 58 years	To describe and compare the meaning, value, and patterns of physical activity and diet among OPLWH and HIV.	Safety concerns in the environment.
Chetty et al. (2022) [15]	South Africa	Phenomenological qualitative study.	16 OPLWH mean age: women 60 years, men 64 years.	To explore OPLWH perceptions of physical activity and exercise.	Pain. HIV-related stigma. Poor health. Safety concerns in the environment.
Voigt et al. (2023) [46]	USA	Secondary data analysis.	100 OPLWH aged 50 years and above.	To assess the physical activity levels among OPLWH and evaluate the relationship with environmental factors.	Safety concerns in the environment.
Neff et al. (2018) [47]	USA	Qualitative study using focus group discussions.	19 older men living with HIV.	To assess the barriers and facilitators to initiating an exercise programme among OPLWH.	Pain. HIV-related stigma. Negative gym experiences. Lack of motivation.
Quigley et al. (2019) [48]	Canada	Qualitative study using semi-structured interviews.	12 OPLWH mean age 56.6 years.	To use the Theoretical Domains Framework to assess barriers and facilitators to participation in exercise among OPLWH.	Poor health and multimorbidity. Lack of motivation. HIV-related stigma.

Source: Researchers.

### 3.3. Outcomes from the Assessment of Risk of Bias

The above studies were of satisfactory quality, and our overall appraisal decision was to include them all in the review. Seven studies were assessed using the qualitative checklist; most (85.7%, n = 6) had two criteria with unclear reporting. Six studies were evaluated using the checklist for analytical cross-sectional studies, and most (66.7%, n = 4) had two of the eight items unclear or not reported. Two studies were appraised using the cohort studies appraisal checklist; in one study, the response was “yes” for seven criteria, and in the other study, six of the responses were “yes”. A summary of the appraisal is shown in Additional File S3.

### 3.4. What Are the Characteristics of OPLWH Experiencing Challenges in Physical Activity Participation?

The studies reviewed included OPLWH with sample sizes that ranged from 10 [44] to 333 [25]. Most (70.6%, n = 12) studies reviewed included more male participants than females. In three studies, more female participants were represented than male participants. In one study [46], there was an equal representation of male and female participants, while in another study [47], only male participants were included.

Most (76.5%, n = 13) studies indicated whether participants had other chronic conditions. In two Canadian studies, all participants had multimorbidity. In the USA, Neff et al. [47] and Voigt et al. [46] indicated that 79% of participants had three or more chronic conditions and 80% had one chronic co-morbidity, respectively. Likewise, Nguyen et al. [40] indicated that all participants had chronic pain, while Duncan et al. [11] recruited only participants with impaired fasting blood glucose levels. In Uganda, it was highlighted that the common co-morbidities were cardiovascular disease, diabetes, and hypertension [22]. In China, Qin et al. [45] stated that the mean number of chronic conditions among participants was 1.3.

The duration of HIV infection of participants in the selected studies was denoted using the mean, median, or median year of diagnosis. Most (35.3%, n = 6) studies included participants who had lived with HIV for 20 years or more. Four (23.5%) studies had participants who had lived with HIV for between 10 and 20 years. Three (17.6%) studies [22,25,45] included participants who had lived with HIV for less than 10 years. One study [16] noted that the mean duration of HIV infection was 15 years for females and 20 years for males. In addition, another study [15] stated that the average HIV infection duration was 8 years for males and 12 years for females.

The majority of studies (94.1%, n = 16) reported that most participants were virally suppressed, and one study indicated that 68.8% of participants were virally suppressed, while Bernard et al. [25] highlighted that 14.4% had detectable levels of HIV. Table 2 summarises the characteristics of participants in the reviewed studies.

**Table 2:** Characteristics of the OPLWH.

Scheme	Percentage of Men/Women	Type and Percentage of Comorbidities	Average/Mean/Median Duration of HIV Infection or Year of HIV Diagnosis
Johs et al. (2019) [16]	86.2% men	Hypertension (34.5%) Diabetes (6.9%) Hyperlipidaemia (20.7%) Depression/anxiety (65.5% Osteoporosis (3.5%)	Mean = 20 years for exercising men, 20 years for non-exercising men, and 15 years for all women
Li et al. (2023) [38]	62.1% men	Not specified	Mean = 16.72 years
Neff et al. (2019) [47]	100.0% men	* Three or more comorbidities (79.0%)	Mean = 19.6 years
Nguyen et al. (2017) [40]	81.5% men	Chronic pain (100.0%)	Mean = 21.3 years
Tatsilong Pambou et al. (2023) [22]	61.0% women	Not specified	HIV duration was not stated; however, 57% of OPLWH had been on ART for less than 7 years.
Derry-Vick et al. (2022) [39]	67.0% men	Depressive symptoms (50.0%), Severe to moderate pain (48.0%) Mild to very mild pain (38.0%)	Mean = 23 years
Qin et al. (2022) [45]	73.0% men	* The mean number of chronic illnesses was 1.3	79% had received an HIV diagnosis within 4 years
Simonik et al. (2016) [41]	64.0% men	Addiction (50.0%) Asthma (35.7%) Cancer (35.7%) Eye disorders (35.7%) Hepatitis C (35.7%) Mental Health conditions (28.6%) Muscle pain (28.6%) Joint pain (28.6%) Hypertension (21.4%) Peripheral neuropathy (14.3%) Arrhythmia (14.3%) Frailty (14.3%) Neurocognitive decline (14.3%)	Median year of diagnosis: 1991
Voigt et al. (2023) [46]	50.0% women and 50.0% men	Cardiac conditions (35%) Respiratory disorders (17.0%) Musculoskeletal conditions (22.0%) Metabolic disorders (11.0%) Mood disorders (43.0%) Sexually Transmitted Infections (21.0%)	Average = 21 years
Chetty et al. (2022) [15]	56.3% men	Not specified	Average = 8 years for men Average = 12 years for women
Duncan et al. (2020) [11]	75.0% men	Impaired fasting blood glucose (100%)	Mean 15.6 years
Homayouni et al. (2021) [44]	80.0% men	Mental health conditions (70.0%), Cardiovascular conditions (60.0%) Bone and joint disorders (40.0%) Diabetes (20.0%) Eye disorders (30.0%) Muscle pain (50.0%)	Median year of diagnosis: 1994
Wright et al. (2021) [31]	68.0% women	Cardiovascular disease (9.0%), Diabetes (26.0%), High cholesterol (88.5%), Chronic kidney disease (9.0%) Tuberculosis (21.5%).	Mean 15.6 years
Quigley et al. (2019) [48]	75.0% men	Not specified	Mean 20 years
Montgomery et al. (2017) [43]	64.0% men	Mental health conditions (55.0%) Bone and joint disorders (45.0%) High cholesterol (36.0%) Neurocognitive decline (36.0%) Addiction (27.0%) Human papilloma virus (27.0%) Peripheral neuropathy (27.0%)	Median year of diagnosis: 2004
Solomon et al. (2021) [42]	64.0% men	Joint pain (45.0%) Mental health conditions (45.0%) High cholesterol (45.0%) Elevated lipid levels (36.0%) Muscle pain (27.0%) Bone and joint disorders (27.0%) Elevated triglycerides (27.0%)	Median year of HIV diagnosis: 2006
Bernard et al. (2020) [25]	57.7% women	Hypertension (21.9%) Arthrosis (15.6%) Migraine (10.2%) Diabetes (6.0%) Hyperlipidaemia (3.6%) Hepatitis B or C (2.7%)	Median = 9 years

* Indicates where the type of comorbidity is not specified. Source: Researchers.

### 3.5. Where Do OPLWH Experience Challenges in Physical Activity Participation?

Articles selected identified OPLWH experiencing physical activity challenges from rural, urban, and peri-urban contexts. One South African study [15] drew its participants from a semi-rural area. Similarly, a Ugandan study [31] explained that participants were selected from an area transitioning into becoming urban. Some studies were conducted in urban areas [11,22,25,46].

The studies reviewed indicated several healthcare and community settings where OPLWH were recruited to join the study. These settings included HIV primary care clinics, community dwellings, medical units, public district hospitals, and AIDS service organisations. Some studies recruited participants from more than one healthcare or community setting.

Most studies indicated that participants were selected from one setting, either a community or a health facility. Of these, one study [11] recruited its participants from HIV clinics and the other two from a district public health facility [15,22]. Additionally, two studies recruited their participants from a university-affiliated HIV clinic [38,39]. Canadian studies recruited participants from the community [44], a specialist hospital [41], and community-based organisations [42]. In the United States, Nguyen et al. [40] recruited participants from an AIDS service organisation, while in Senegal and Côte d’Ivoire, respondents were selected from urban HIV clinics [25].

Two studies highlighted the use of two different settings to recruit participants. Johs et al. [16] recruited from the community and university-managed HIV clinics, and Voigt et al. [46] selected participants from AIDS service organisations and an HIV clinic. A Canadian study recruited participants from numerous community settings and AIDS service organisations [48].

### 3.6. What Are the Challenges Experienced by OPLWH in Physical Activity Participation?

The review identified individual-related challenges and community or environmental-related challenges.

#### 3.6.1. Individual-Related Challenges to Physical Activity Participation

The review found four individual-related physical activity challenges. These were pain, depression, poor physical health, and lack of motivation.

Individual Challenges Relating to OPLWH’S Health

Studies included in this review included OPLWH who lived with other chronic conditions, as shown in Table 2; therefore, three of the challenges (pain, depression, and poor physical health/multimorbidity) related to the health of the OPLWH.

- Poor Physical Health and Multimorbidity

Most studies [15,16,22,25,40,41,42,43,44,48] illustrated how poor physical health and multimorbidity affected physical activity. In the USA, fatigue and side effects of medication associated with chronic illnesses, such as hypertension, diabetes, osteoporosis, and depression/anxiety, affect physical activity participation [16]. A United States participant noted, “*I got sick, and I had a lot of problems with my legs, and I wasn’t really able to walk*” [16]. Other USA participants who all had chronic pain perceived that having poor balance and being overweight characterised poor physical health [40]. A South African participant summed up the two issues of fatigue and perceptions of being overweight by noting, “*But the issue is that I got tired*” … “*My body is too heavy; it’s failing me*” [15]. Moreover, the South African participants noted that poor physical health was characterised by pain [15]. Pain was also described by OPLWH who had comorbidities (joint pain, muscle pain, bone and joint disorders) and required special exercise considerations [42].

Canadian OPLWH with multimorbidity like mental health conditions, cardiovascular conditions, bone and joint disorders, eye disorders, and diabetes, described the effects of poor physical health as “*episodic*” [44]. Other Canadian studies [41,43,48], which also included participants with joint pain and mental health conditions, corroborated the fluctuating poor physical health. This may emanate from side effects of medication, comorbidities, and injuries [16,40]. A Canadian participant explained the episodic nature of poor physical health: “*It’s not possible to have a strict routine [exercise] because every day is a different day when you’re sick or not sick*” [41]. Furthermore, the West African study by Bernard et al. [25] also found that lower physical activity (walking gait, balance, and lower limb strength) was associated with OPLWH who had diabetes, hypertension, and abdominal obesity.

- Pain experienced by OPLWH

The issue of pain was further reiterated by five studies [15,39,40,41,47] from South Africa, the USA, and Canada, which found that pain affected physical activity participation. In semi-rural South Africa, one participant noted that the only thing that distanced them from exercise was back pain [15]. Other pain sites revealed were joints and muscles, which affected walking [15]. Leg pain was also noted by older men in the USA who previously ran but stopped because of ankle joint pain [47]. Chronic pain was also described in the USA and Canada [40,42]. United States participants with chronic pain feared engaging in physical activity because the pain would be exacerbated; one participant described, “*My major pain is in my knee, and I’m just afraid of twisting wrong*” [40]. Canadian participants explained how pain varied daily. One participant in a study that included OPLWH with chronic pain noted, “*My body is aching and sore…it’d be hard to exercise*” [41]. These qualitative studies [15,40,41,47] are corroborated by a quantitative study, which revealed that OLPWH had a slower walking gait, decreased hand grip strength, and self-reported lower physical function with increased pain levels [39].

- Depression among OPLWH

Five studies [22,38,41,45,48] from the USA, Cameroon, Canada, and China found that physical activity is affected by depressive symptoms. In the United States, lower levels of depressive symptoms, higher socio-economic status, and younger age were associated with higher levels of physical activity [38]. Similarly, in Cameroon, lower physical activity levels were associated with the psychological and spiritual domains of the WHO quality of life questionnaire [22]. This WHO psychological domain included depressive symptoms, anxiety, and hopelessness [22]. Likewise, a Chinese study found that OPLWH with lower physical activity levels had higher levels of depressive symptoms compared to HIV-negative older people [45].

Findings from Canada clarify how depressive symptoms adversely affect physical activity participation by noting that OPLWH with co-morbid depression failed to sustain exercise for an extended period because of a “*loss of interest*” [41]. Paradoxically, depressive symptoms were eased by physical activity [41]. OPLWH also revealed that anxiety and depression result in procrastinating physical activity due to not being in an emotionally good space [48].

Individual Challenges Related to the OPLWH’s Agency

The study found that the lack of motivation affected the OPLWH’s agency to perform physical activity. Agency refers to the individual’s capacity or power [49] in this context, to engage in physical activity.

- Lack of motivation

Five studies from the USA [16,47], Canada [44,48], and the UK [11] described how the lack of motivation emanating from ageing, an HIV diagnosis with associated fatigue, and a lack of social support affected physical activity participation. In the USA, the lack of motivation emanated from an HIV diagnosis, with associated fatigue and old age [16,47]. One USA participant noted, “*Once diagnosed, I kind of lost interest*” [47]. In the UK, participants lacked motivation to exercise as they also believed it was futile due to an HIV diagnosis and probable development of type 2 diabetes as they age: “*As you get older and you’re on these [HIV] drugs, this [diabetes] is just going to happen. There’s nothing you can do*” [11]. Canadian participants illustrated that the lack of motivation was associated with individual exercise. One participant revealed, “*If I do it on my own, it’s not the same*” [44]. Another Canadian study found that lack of motivation affected OPLWH’s intention to exercise [48].

#### 3.6.2. Challenges Experienced in the Community or Environment

The OPLWH experienced challenges in physical activity participation from the community and the environment. These were HIV-related stigma, negative experiences at the gym, and safety concerns in the environment.

- HIV-Related Stigma

Four studies from the UK [11], Canada [44,48], and South Africa [15] described the issue of HIV-related stigma. South African and UK participants shared how HIV-related stigma originated from forced disclosure [11,15]. In the UK, there was a perception that weight loss from physical activity would lead to forced HIV disclosure or indicate the presence of HIV opportunistic infections, which were stigmatised [11]. Comparably, South African participants explained that group exercise was problematic because people could identify them as PLWH, yet they were not prepared to disclose their HIV status. A South African participant noted, “*People outside, they will be able to identify that the people who are exercising here are the people who are HIV-positive, this is something I am not comfortable talking about*” [15]. Canadian participants agreed with the South African participants on how judgement from community members would discourage them from exercising: “*I guess the judgement…*” [44]. However, the same Canadian participant further explained that among themselves as OPLWH, there was no stigma, and they did not feel judged, “*whereas here it didn’t feel that way*” [44]. Another Canadian study described how HIV-related stigma was expressed in different ways in the community; sometimes overtly, like, “*Oh, I don’t want to meet you because you’re HIV positive… So yeah, there are very direct experiences of stigma*” [48].

- Negative gym experiences

Negative experiences at the gym were described in six studies [11,16,40,41,44,47]. These negative experiences were founded on poor relations with other patrons or gym instructors and physical aspects of the gym (machinery and infrastructure). Concerning the physical aspects of the gym, United States participants shared experiences of using technologically advanced gym machinery they were unfamiliar with: “*There are technological advances that have happened since I spent any time in a gym*” [16]. Canadian participants shared their experiences of inadequate space, resulting in a reluctance to exercise: “*We’ve got a small gym here, and when there’s too many people, it’s a bit uncomfortable*” [44]. Also, in Canada, participants highlighted the gym infrastructure issues faced by OPLWH with multimorbidity and disability [41]. From their shared experiences, stairs and the lack of lockers accessible to people in wheelchairs were deterrents. One participant reflected, “*Cause there’s no lockers for people with wheelchairs, the gym has a staircase to get into the aqua fit pool…*” [41].

Regarding the relational issues at the gym, participants in Canada and the United States described connotations of HIV-related stigma, such as alienation. Canadian participants shared how disclosure affected relations with patrons who are HIV-negative when there are no exercise programmes specific to PLWH. One participant noted, *“You get somebody that doesn’t know and doesn’t like it. You’re screwed. Alienated*” [41]. Similarly, in the USA, participants perceived that they could not meet the physical demands of exercise programmes that were not specific to OPLWH [40]. Without exercise programmes exclusive to OPLWH, participants were deterred as they did “*not feel safe*” in “*non-positive*” places [40]. Another USA study found that OPLWH were uncomfortable with their “*old body*” images in the gym environment compared to other patrons, whom they felt were in a “*runway show*” [47]. In the UK, participants were similarly concerned about other patrons’ thoughts of their body images due to HIV-associated lipodystrophy; one female participant noted, “*Every time I go to the gym, people think I’m pregnant because of the lipodystrophy*” [11].

- Safety concerns in the environment

Two environmental safety aspects affecting outdoor physical activity were described by participants from the USA [46], South Africa [15], and Uganda [31]: crime and traffic safety. South African and Ugandan participants were concerned about crime, especially in the dark. One Ugandan participant shared their experience: “*So while returning home, armed thugs took my phone and the car*” [31]. Describing the Ugandan setting, Wright et al. [31] explain that most participants engaged in farming activities as a form of physical activity. The South African participants who expressed that crime was a problem resided in the semi-rural area [15]. A quote from one South African participant reads, *“I also want to run in a safe area. I don’t run in dangerous places where it’s dark*” [15]. Although crime affects outside physical activity, it is acknowledged that this concern may not be related to HIV or ageing and can be experienced by anyone [31]. In New York City (USA), OPLWH were concerned about traffic hazards as motorists failed to obey speed limits in the area [46].

### 3.7. The Conceptual Diagram of Study Findings

From the study findings, the following conceptual figure, which outlines the relationship between the individual and the community/environmental factors affecting physical activity participation among OPLWH, was derived.

**Figure 2:**
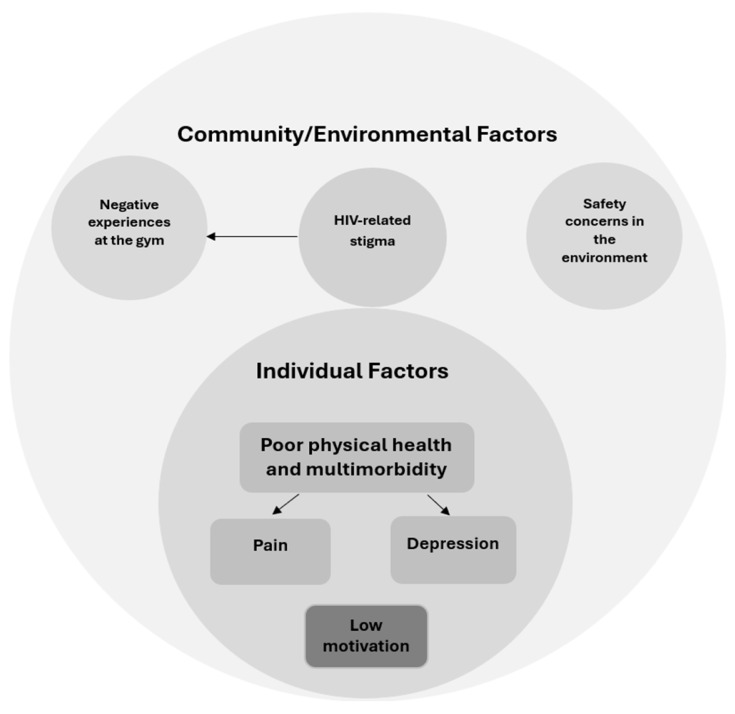
The conceptual diagram of study findings. Source: researchers.

Figure 2 shows that OPLWH live with multimorbidity, which may affect physical activity due to fatigue and drug side effects. These chronic conditions also include pain and depression, which affect the ability to participate in physical activity. At the individual level, OPLWH may also have low motivation to exercise. An individual older person living with HIV may also live in a community which may not be safe for outdoor physical activity or may be marked by HIV-related stigma, which can also be experienced at the gym.

## 4. Discussion

The challenges experienced by OPLWH in performing physical activity include poor physical health/multimorbidity, pain, depression, lack of motivation, HIV-related stigma, negative experiences at the gym, and safety concerns in the environment.

The studies included in the scoping review were mostly from the USA, which is concerning, as most (80%) OPLWH reside in sub-Saharan Africa and East Africa [27]. This lack of representation of studies from sub-Saharan Africa mirrors the underrepresentation of African studies at a global scale [50], possibly due to systematic challenges such as the lack of funding for research in Africa [51]. Although most studies are from areas outside sub-Saharan Africa, this also illustrates the pertinence of the issue across different geographical settings, including developed countries where a more significant proportion of OPLWH, compared to younger PLWH, reside [27]. Studies reviewed from sub-Saharan Africa included those from South Africa, Uganda, Côte d’Ivoire, Senegal, and Cameroon, and comprised one study each; this also confirms previous findings by Bernard et al. [25], who noted the paucity of literature from sub-Saharan Africa. The underrepresentation of studies from sub-Saharan Africa may limit the application of findings in this context. For example, the study found that OPLWH had negative experiences at the gym. However, the studies reviewed were from countries outside sub-Saharan Africa. This lack of representation could imply a need for further studies on OPLWH experiences in performing physical activity in the gym. In addition, it is recommended that further studies should compare challenges in physical activity participation among the different contexts (Africa, Europe, and North America), given that the issue of negative experiences at the gym was only described in contexts outside Africa.

The review found that HIV-related stigma hindered physical activity participation. Stigma, like depression, was an issue shared across several geographic settings by studies from the UK, USA, South Africa, and Canada, where samples were drawn from semi-rural and urban communities. This inclusion of different geographical locations and settlements illustrates the universality of HIV-related stigma in communities. This confirms the findings of other studies, which note the ubiquity of HIV-related stigma and its effect on all aspects of the HIV care continuum [52]. This finding also confirms a recent study conducted in the UK, which notes that although there is a change in how stigma manifests, there is a limited change in public knowledge and attitudes towards HIV [53]. Therefore, because of this continued impact of HIV-related stigma, health workers must develop community-based exercise programmes designed for the needs of OPLWH and inclusive gym environments where anti-stigma education is provided [54,55].

The study highlighted how safety concerns in the environment impede physical activity. This finding was drawn from studies conducted in Uganda [31], South Africa [15], and the USA [46]. However, there was a difference in the type of concern; in the African studies, the OPLWH were concerned with crime in the communities, especially in the dark, while in the USA study, the concern was traffic hazards. The results from South Africa and Uganda highlight the issue of rural crime, as both studies were conducted in similar semi-rural contexts. This confirms the findings of the systematic review undertaken on rural crime in Africa, which revealed that some of the concerns about crime were related to environmental issues and safety concerns among rural residents [56]. Nonetheless, the differences in safety concerns in the environment between the two settings may warrant context-specific studies to conclude which aspects of the physical environment adversely affect outside physical activity among OPLWH.

The effect of pain on physical activity is documented in other studies [18,19]. This scoping review provides further confirmation that there is a need to consider other chronic conditions among OPLWH, given that some studies [41,47] in this review included OPLWH with multimorbidity and outlined the pain issue. This may confirm the findings by Karris et al. [20], who noted that among OPLWH, pain may emanate from other age-related chronic conditions such as osteoarthritis. Moreover, Derry-Vick et al. [39] reported that among OPLWH, the presence of pain is associated with depressive symptoms, which are likely to affect physical activity participation. To confirm the issue of other chronic conditions affecting physical activity participation, most studies included in this review described the problem of multimorbidity. Therefore, this finding was not unexpected, more so as some studies [41,44] included only OPLWH with multimorbidity. However, this effect of multimorbidity on physical activity might warrant special consideration of OLPWH in promoting physical activity, given the episodic nature of poor physical health.

### Strengths and Limitations

The study had several limitations; firstly, there was inadequate representation of studies from sub-Saharan Africa, where most OPLWH reside. Secondly, the study was restricted to English peer-reviewed articles, which may have left out data from grey literature and articles published in other languages. Thirdly, there could have been potential bias in selecting the three databases and excluding other databases. Nonetheless, the main strength of the study was the inclusion of articles that enabled the saturation of themes for each challenge described.

## 5. Conclusions

The scoping review described the challenges OPLWH faced in engaging in physical activity. The OPLWH, who described these challenges, were drawn from studies where men comprised the larger proportion of participants, and where most OPLWH were virally suppressed. The individual-related challenges included pain, depression, a lack of motivation, and poor physical health/multimorbidity. Community- and environment-level challenges included HIV-related stigma, negative experiences at the gym, and personal safety concerns. From this review, it is recommended that additional studies on gym experiences among OPLWH in sub-Saharan Africa should be conducted to add knowledge to this understudied phenomenon. It is also recommended that anti-stigma education should be provided as part of community-based physical activity interventions.

## Data Availability

Data supporting reported results can be requested from the corresponding author, N.M.

## Supplementary Materials

The following supporting information can be downloaded at: https://www.mdpi.com/article/10.3390/ijerph22101513/s1, Additional File S1: The database search strategy; Additional File S2: The data extraction tool; Additional File S3: The outcome of the risk of bias assessment; Additional File S4: Preferred Reporting Items for Systematic reviews and Meta-Analyses extension for Scoping Reviews (PRISMA-ScR) Checklist. Reference [57] are cited in the supplementary materials.

## Abbreviations

The following abbreviations are used in this manuscript:

OPLWH	Older People Living With HIV
HIV	Human Immunodeficiency Virus
PRISMA	Preferred Reporting
ART	Antiretroviral Therapy
USA	United States of America
UK	United Kingdom
AIDS	Acquired Immunodeficiency Syndrome
WHO	World Health Organization
PCC	Population Concept and Context
LMIC	Low-to-Middle-Income Countries
GAPPA	Global Action Plan on Physical Activity
