# Supplementary material for: Challenges to Physical Activity Participation Among Older People Living with HIV: Scoping Review
Source: Int J Environ Res Public Health. 2025 Oct 2;22(10):1513. doi: 10.3390/ijerph22101513 (PMC12563587; doi:10.3390/ijerph22101513)
Supplement: Supplementary file 1 [file ijerph-22-01513-s001.zip › Additional File A.pdf]

ADDITIONAL FILE A: THE SEARCH STRATEGY

Title: Challenges to Physical Activity Participation Among Older People Living with HIV: Scoping Review.

**The PubMed Search Strategy**		
**The full search strategy**	7,(((#1) AND (#2)) AND (#3)) AND (#5),,in the last 10 years,"((""exercise""[MeSH Terms] OR ""exercise""[All Fields] OR (""physical""[All Fields] AND ""activity""[All Fields]) OR ""physical activity""[All Fields] OR (""walked""[All Fields] OR ""walking""[MeSH Terms] OR ""walking""[All Fields] OR ""walks""[All Fields]) OR ""excercise""[All Fields] OR ""motor activity""[All Fields] OR ""physical excercise""[All Fields] OR ((""physical examination""[MeSH Terms] OR (""physical""[All Fields] AND ""examination""[All Fields]) OR ""physical examination""[All Fields] OR ""physical""[All Fields] OR ""physically""[All Fields] OR ""physicals""[All Fields]) AND (""recreation""[MeSH Terms] OR ""recreation""[All Fields] OR ""recreations""[All Fields] OR ""recreational""[All Fields] OR ""recreator""[All Fields] OR ""recreators""[All Fields])) OR (""exertion""[All Fields] OR ""exertional""[All Fields] OR ""exertions""[All Fields]) OR (""movement""[MeSH Terms] OR ""movement""[All Fields] OR ""movements""[All Fields] OR ""movement s""[All Fields]) OR (""sport s""[All Fields] OR ""sports""[MeSH Terms] OR ""sports""[All Fields] OR ""sport""[All Fields] OR ""sporting""[All Fields]) OR ""physical training""[All Fields] OR ""working out""[All Fields] OR (""workout""[All Fields] OR ""workouts""[All Fields]) OR (""aerobic""[All Fields] OR ""aerobically""[All Fields] OR ""bacteria, aerobic""[MeSH Terms] OR (""bacteria""[All Fields] AND ""aerobic""[All Fields]) OR ""aerobic bacteria""[All Fields] OR ""aerobe""[All Fields] OR ""aerobes""[All Fields] OR ""exercise""[MeSH Terms] OR ""exercise""[All Fields] OR ""aerobics""[All Fields]) OR ""sport activity""[All Fields] OR (""excercise""[All Fields] AND (""stress""[All Fields] OR ""stressed""[All Fields] OR ""stresses""[All Fields] OR ""stressful""[All Fields] OR ""stressfulness""[All Fields] OR ""stressing""[All Fields])) OR ""physical movement""[All Fields] OR ""physical effort""[All Fields] OR ""physical function""[All Fields] OR ""bodily movement""[All Fields] OR (""gymnast""[All Fields] OR ""gymnast s""[All Fields] OR ""gymnastics""[MeSH Terms] OR ""gymnastics""[All Fields] OR ""gymnastic""[All Fields] OR ""gymnasts""[All Fields]) OR ((""movement""[MeSH Terms] OR ""movement""[All Fields] OR ""movements""[All Fields] OR ""movement s""[All Fields]) AND (""human body""[MeSH Terms] OR (""human""[All Fields] AND ""body""[All Fields]) OR ""human body""[All Fields] OR	**Results**

	""body""[All Fields])) OR (""fitness""[All Fields] OR ""fitnesses""[All Fields]) OR ""fitness regimen""[All Fields] OR (""strenous""[All Fields] AND (""activable""[All Fields] OR ""activate""[All Fields] OR ""activated""[All Fields] OR ""activates""[All Fields] OR ""activating""[All Fields] OR ""activation""[All Fields] OR ""activations""[All Fields] OR ""activator""[All Fields] OR ""activator s""[All Fields] OR ""activators""[All Fields] OR ""active""[All Fields] OR ""actived""[All Fields] OR ""actively""[All Fields] OR ""actives""[All Fields] OR ""activities""[All Fields] OR ""activity s""[All Fields] OR ""activitys""[All Fields] OR ""exercise""[MeSH Terms] OR ""exercise""[All Fields] OR ""activity""[All Fields])) OR ""physical labour""[All Fields] OR ((""movement""[MeSH Terms] OR ""movement""[All Fields] OR ""movements""[All Fields] OR ""movement s""[All Fields]) AND (""muscle, skeletal""[MeSH Terms] OR (""muscle""[All Fields] AND ""skeletal""[All Fields]) OR ""skeletal muscle""[All Fields] OR (""skeletal""[All Fields] AND ""muscles""[All Fields]) OR ""skeletal muscles""[All Fields])) OR ""exercise routine""[All Fields] OR ""activity level""[All Fields]) AND (""aged""[MeSH Terms] OR ""aged""[All Fields] OR ""aged over 50""[All Fields] OR (""older""[All Fields] OR ""olders""[All Fields]) OR (""geriatric""[All Fields] OR ""geriatrics""[MeSH Terms] OR ""geriatrics""[All Fields]) OR (""ageism""[MeSH Terms] OR ""ageism""[All Fields]) OR (""pensionable""[All Fields] OR ""pensioned""[All Fields] OR ""pensioner""[All Fields] OR ""pensioner s""[All Fields] OR ""pensioners""[All Fields] OR ""pensioning""[All Fields] OR ""pensions""[MeSH Terms] OR ""pensions""[All Fields] OR ""pension""[All Fields]) OR ""senior citizens""[All Fields] OR (""aged""[MeSH Terms] OR ""aged""[All Fields] OR ""elderly""[All Fields] OR ""elderlies""[All Fields] OR ""elderly s""[All Fields] OR ""elderlys""[All Fields]) OR ""middle aged""[All Fields] OR ""elderly population""[All Fields] OR ""older adults""[All Fields] OR ""old people""[All Fields] OR ""senior generation""[All Fields] OR ""retired people""[All Fields] OR (""retiree""[All Fields] OR ""retirees""[All Fields])) AND (""hiv""[MeSH Terms] OR ""hiv""[All Fields] OR (""hiv""[MeSH Terms] OR ""hiv""[All Fields] OR (""human""[All Fields] AND ""immunodeficiency""[All Fields] AND ""virus""[All Fields]) OR ""human immunodeficiency virus""[All Fields]) OR (""acquired immunodeficiency syndrome""[MeSH Terms] OR (""acquired""[All Fields] AND ""immunodeficiency""[All Fields] AND ""syndrome""[All Fields]) OR ""acquired immunodeficiency syndrome""[All Fields] OR ""aids""[All Fields]) OR (""acquired immunodeficiency syndrome""[MeSH Terms] OR (""acquired""[All Fields] AND ""immunodeficiency""[All Fields] AND ""syndrome""[All Fields]) OR ""acquired immunodeficiency syndrome""[All Fields])) AND (""challenge""[All Fields] OR ""challenged""[All Fields] OR ""challenges""[All Fields] OR	

	""challenging""[All Fields] OR (""difficulties""[All Fields] OR ""difficulty""[All Fields]) OR (""complaint""[All Fields] OR ""complainted""[All Fields] OR ""complaints""[All Fields]) OR (""barrier""[All Fields] OR ""barrier s""[All Fields] OR ""barriers""[All Fields]) OR ""misgivings""[All Fields] OR (""reluctance""[All Fields] OR ""reluctances""[All Fields] OR ""reluctant""[All Fields]) OR (""reluctance""[All Fields] OR ""reluctances""[All Fields] OR ""reluctant""[All Fields]) OR (""uncertainty""[MeSH Terms] OR ""uncertainty""[All Fields] OR ""uncertainties""[All Fields]) OR (""reservation""[All Fields] OR ""reservations""[All Fields]) OR ((""attitude""[MeSH Terms] OR ""attitude""[All Fields] OR ""attitudes""[All Fields] OR ""attitude s""[All Fields]) AND (""exercise""[MeSH Terms] OR ""exercise""[All Fields] OR ""exercises""[All Fields] OR ""exercise therapy""[MeSH Terms] OR (""exercise""[All Fields] AND ""therapy""[All Fields]) OR ""exercise therapy""[All Fields] OR ""exercising""[All Fields] OR ""exercise s""[All Fields] OR ""exercised""[All Fields] OR ""exerciser""[All Fields] OR ""exercisers""[All Fields])) OR (""fear""[MeSH Terms] OR ""fear""[All Fields]))) AND (y_10[Filter])",501,02:37:26,2025/01/23	
**The Google Scholar search strategy**		
	"physical activity" OR walking OR exercise OR "motor activity" OR "physical exercise" OR "physical recreation" OR exertion OR movement OR sport OR play OR "physical training" OR workout OR "physical fitness" OR "working out" OR aerobics OR "sport activity" OR "exercise stress" OR "physical movement" OR "physical effort" OR "physical function" OR "bodily movement" OR gymnastics OR "movement of the body" OR "fitness regimen" OR "strenuous activity" OR "physical labour" OR "movement by skeletal muscles" OR "exercise routine" OR "activity level" AND difficulties OR challenges OR complaints OR barriers OR misgivings OR reluctant OR reservations OR "attitude to exercise" OR fear AND aged OR "aged over 50" OR geriatrics OR ageism OR pensioners OR "senior citizens" OR elderly OR "middle aged" OR "elderly population" OR "older adults" OR "old people" OR "senior generation" OR "retired people" OR retirees AND "human immunodeficiency virus" OR HIV OR AIDS OR "acquired immunodeficiency syndrome"	
**The Scopus Database search strategy**		
	(HIV OR human immunodeficiency virus OR AIDS OR acquired immunodeficiency syndrome) AND (challenges OR complaints OR barriers OR misgivings OR reluctant OR uncertainties OR reservations OR "attitude to exercise" OR fear) AND (aged OR "aged over 50" OR geriatrics OR ageism OR pensioners OR "senior citizens" OR elderly OR "middle aged" OR "elderly population" OR "older adults" OR "old people" OR "senior generation" OR "retired people" OR retirees) AND ("physical activity" OR walking OR exercise OR "motor activity" OR	

	"physical exercise" OR "physical recreation" OR exertion OR movement OR sport OR play OR "physical training" OR workout OR "physical fitness" OR training OR "working out" OR aerobics OR "sport activity" OR "exercise stress" OR "physical movement" OR "physical effort" OR "physical function" OR "bodily movement" OR gymnastics OR "movement of the body" OR "fitness regimen" OR exertion OR "strenuous activity" OR "physical labour" OR "movement by skeletal muscles" OR "exercise routine" OR "activity level" AND PUBYEAR > 2015 AND PUBYEAR < 2025) AND (LIMIT-TO (EXACTKEYWORD , "Human")) AND (LIMIT-TO (DOCTYPE , "ar")) AND (LIMIT-TO (SUBJAREA , "MEDI") OR LIMIT-TO (SUBJAREA , "NURS") OR LIMIT-TO (SUBJAREA , "HEAL") OR LIMIT-TO (SUBJAREA , "SOCI") OR LIMIT-TO (SUBJAREA , "NEUR")) AND (LIMIT-TO (LANGUAGE , "English"))	
