# Supplementary material for: Challenges to Physical Activity Participation Among Older People Living with HIV: Scoping Review
Source: Int J Environ Res Public Health. 2025 Oct 2;22(10):1513. doi: 10.3390/ijerph22101513 (PMC12563587; doi:10.3390/ijerph22101513)
Supplement: Supplementary file 1 [file ijerph-22-01513-s001.zip › Additional File C.pdf]

ADDITIONAL FILE C

Title: Challenges to Physical Activity Participation Among Older People Living with HIV: Scoping Review.

1. JBI Critical Appraisal Checklist for Qualitative Research

		Congruency between philosophical perspective and methodology	Congruency between research methodology and the research question	Congruency between research methodology and data collection methods	Congruency between research methodology and data analysis methods	Congruency between research methodology and the interpretation of results	Statement locating the researcher culturally or theoretically	Influence of the researcher on the research, and vice-versa, addressed	Participants, and their voices, adequately represented	Research ethical according to current criteria or, for recent studies, and is there evidence of ethical approval by an appropriate body	The conclusions drawn in the research report flow from the analysis, or interpretation, of the data	Overall appraisal decision
Chetty et al. (2022)	Yes	X	X	X	X	X		X	X	X	X	Include
	No											
	Unclear						X					
	N/A											
Homayouni et al (2021)	Yes	X	X	X	X	X			X	X	X	Include
	No											
	Unclear						X	X				
	N/A											
Johs et al. (2019)	Yes	X	X	X	X	X			X	X	X	Include
	No											
	Unclear						X	X				
	N/A											
	Yes	X	X	X	X	X			X	X	X	Include

Neff et al. (2019)	No											
	Unclear						X	X				
	N/A											
Nguyen et al. (2017)	Yes	X	X	X	X	X			X	X	X	Include
	No											
	Unclear						X	X				
	N/A											
Quigley et al. (2019)	Yes	X	X	X	X	X			X	X	X	Include
	No											
	Unclear						X	X				
	N/A											
Simonik et al. (2016)	Yes	X	X	X	X	X		X	X	X	X	Include
	No											
	Unclear						X					
	N/A											
Montgomery et al. (2017)	Yes	X	X	X	X	X	X		X	X	X	Include
	No											
	Unclear							X				
	N/A											
Solomon et al. (2021)	Yes	X	X	X	X	X			X	X	X	Include
	No											
	Unclear						X	X				
	N/A											

2. JBI Critical Appraisal for Analytical Cross-sectional Studies

		Criteria for inclusion in the sample clearly defined	Study subjects and the setting described in detail	Exposure measured in a valid and reliable way	Objective, standard criteria used for measurement of the condition	Confounding factors identified	Strategies to deal with confounding factors stated	The outcomes measured in a valid and reliable way	Appropriate statistical analysis used	Overall appraisal decision
Derry-Vick et al. (2022)	Yes	X	X	X	X			X	X	Include
	No						X			
	Unclear					X				
	N/A									
Li et al. (2023)	Yes	X	X	X	X			X	X	Include
	No						X			
	Unclear					X				
	N/A									
Pambou et al. (2023)	Yes	X	X	X	X			X	X	Include
	No						X			
	Unclear					X				
	N/A									
Qin et al. (2022)	Yes	X	X	X	X	X	X	X	X	Include
	No									
	Unclear									
	N/A									
Voight et al. (2023)	Yes	X	X	X	X			X	X	Include
	No						X			
	Unclear					X				
	N/A									
Bernard et al. (2020)	Yes	X	X	X	X	X	X	X	X	Include
	No									
	Unclear									
	N/A									

3. JBI Checklist for Cohort Studies

		Two groups similar and recruited from the same population	Exposures measured similarly to assign people to both exposed and unexposed groups	The exposure measured in a valid and reliable way	Confounding factors identified	Strategies to deal with confounding factors stated	Groups/participants free of the outcome at the start of the study (or at the moment of exposure)	Outcomes measured in a valid and reliable way	Follow up time reported and sufficient to be long enough for outcomes to occur	Follow up complete, and if not, were the reasons to loss to follow up described and explored	Strategies to address incomplete follow up utilized	Appropriate statistical analysis used	Overall appraisal decision
Duncan et al. (2020)	Yes			X			X	X	X	X		X	Include
	No					X							
	Unclear				X								
	N/A	X	X								X		
Wright et al. (2021)	Yes	X	X	X				X	X	X		X	Include
	No					X							
	Unclear				X								
	N/A						X				X		
